# Associations between Leisure Preferences, Mindfulness, Psychological Capital, and Life Satisfaction

**DOI:** 10.3390/ijerph19074121

**Published:** 2022-03-30

**Authors:** Aiste Dirzyte, Aleksandras Patapas, Aidas Perminas

**Affiliations:** 1 Institute of Psychology, Mykolas Romeris University, 08303 Vilnius, Lithuania; 2 Faculty of Creative Industries, Vilnius Gediminas Technical University, 10223 Vilnius, Lithuania; 3 Institute of Public Administration, Mykolas Romeris University, 08303 Vilnius, Lithuania; patapas@mruni.eu; 4 Department of Psychology, Vytautas Magnus University, 44248 Kaunas, Lithuania; aidas.perminas@vdu.lt

**Keywords:** mindfulness, psychological capital, life satisfaction, leisure preferences

## Abstract

This study intended to explore which leisure preferences contribute to mindfulness, psychological capital, and life satisfaction and assess whether mindfulness, psychological capital, and life satisfaction are associated with different leisure preferences. This study applied the Satisfaction with Life Scale (SWLS), the Psychological Capital Questionnaire (PCQ-12), the Mindful Attention Awareness Scale (MAAS), and the instrument to evaluate the prevalence of leisure preferences. A sample consisted of 586 participants, 104 males and 478 females. The mean age of participants was 42.06, SD = 13.29. The results show that respondents who did not spend free time watching television scored higher on life satisfaction, mindfulness, and psychological capital. Participants who preferred attending events scored higher on life satisfaction and psychological capital. Participants who preferred spending time with family as a leisure preference scored significantly higher on life satisfaction, mindfulness, and psychological capital, including PsyCap overall, PsyCap work, PsyCap relationship, and PsyCap health. The findings also reveal that time spent with family is significantly associated with life satisfaction. Besides, males’ life satisfaction was significantly associated with time spent in nature, while females’ satisfaction was associated with spending time with family and participating in events. Males’ mindfulness was significantly associated with book reading, and females’ mindfulness was associated with not watching television. Males’ psychological capital was significantly associated with spending time with family and book reading, and females’ psychological capital was associated with not watching television but spending time with family, participating in events, and spending time in nature. The findings also showed that mindfulness mediated the link between watching television and life satisfaction, and psychological capital mediated links between spending time with family, participating in events, and life satisfaction. The findings demonstrate that life satisfaction is also significantly associated with spending time with family as a leisure preference. This study also revealed a significant negative association between age and spending time with friends or family, evidencing the possible loneliness of elderly respondents. Due to limitations of this study, including sample size and characteristics, cultural context, and research design, the research findings would preferably be regarded thoughtfully.

## 1. Introduction

Due to the COVID-19 pandemic, people worldwide faced tense life challenges, including restrictions in leisure activities. The global lockdown has affected leisure choices, resulting in reconsidering “how, where, and when leisure takes place” [1]. Leisure, which by its Latin origin “licere” means “to be permitted” or “to be free”, gained a new perspective of somewhat limited time spending possibilities linked to leisure nostalgia [2].

The importance of daily leisure on quality of life could be based on research evidencing that psychological wellbeing increases immediately during vacation, peaks on the eighth vacation day, and rapidly returns to baseline level within the first week of work resumption [3]. As the pandemic has restricted vacation possibilities, new knowledge on the effects of daily leisure preferences on life satisfaction is needed. 

### 1.1. Leisure Preferences

Various research has documented that leisure is one of the essential life quality domains and core ingredients of wellbeing [4]. Leisure activities reflect particular profiles corresponding to numerous variables, including age [5,6,7], health [8,9,10], goals and personality traits [11], gender [12,13], cultural contexts [14,15,16,17,18], emotions [19,20,21], intrinsic motivational orientation [22], among others. 

Newman et al. proposed that the multidimensional construct of leisure encompasses structural (amount of time spent outside of work, the number of activities viewed as leisure) and subjective (sense of leisure involvement, positive feelings) aspects [4]. Subjective leisure is not necessarily tied to a particular activity but might be linked to a particular flow [23] or relaxation [24] in environments where one can feel absorbed by the present moment, free from obligations. 

Research indicates that leisure preferences, allowing pleasant absorption in the present moment, might have beneficial or detrimental effects. Such leisure preferences as “recreational marihuana” [25] consumed in the user’s leisure time to relax or enhance positive affect, might temporally disengage from stress, but the long–term consequences might be rather far from the desired. On the other hand, some leisure preferences are beneficial for the user’s health and wellbeing, including meditation [9] or physical activity [26,27,28], or spending time in nature [14,29]. However, excessive engagement in some types of “user-friendly” leisure preferences might also result in wellbeing and health-related challenges [30].

Based on previous research linking various leisure activities to psychological wellbeing, we targeted several types of leisure preferences, namely, (1) time spent with family, (2) time spent with friends, (3) time spent in nature, (4) participating in events, (5) watching TV, and (6) book reading. Research suggests that these leisure preferences might signify certain qualities of a user.

(1) Time spent with family. As a leisure preference, time spent with family indicates several user attributes. Firstly, for the user, family is a value. Research indicates that family and leisure values are positively linked to life satisfaction in all five regions: the West, Latin America, the Asian-Confucian region, ex-Communist Eastern Europe, and the Communist countries of China and Vietnam [19]. However, not every user prefers family for leisure purposes [31,32,33,34,35,36]. If the time spent with family is preferred as a leisure choice, it suggests that being with family can satisfy the user’s basic psychological needs of autonomy, competence, and relatedness [11,37]. From a Hsiao Yao (逍遙) perspective, which stems from Taoism’s Chuang Tzu’s philosophy, leisure is a carefree state of being or happiness [38]. It means that for some people, spending time with family can satisfy needs for relaxation, safety [39,40], or affiliation [4]. Next, research suggests that family leisure satisfaction is the strongest predictor of family functioning and satisfaction with family life [32,41]. Consequently, family as a leisure preference might significantly predict overall life satisfaction.
(2) Time spent with friends. If a user enjoys leisure with friends, it can be assumed that one also scores high on extraversion and can establish and maintain strong social bonds based on mutual trust, compassion, and understanding. In conceptual research on “The social nature of leisure involvement”, Kyle and Chick proposed that leisure is a group phenomenon, and social circles, including friends, highly influence participation in recreation [42]. Next, they noted that the social organization of leisure is characterized by people interacting with others due to mutual tastes and a sense of belonging: “building memories of past experiences shared with family and friends were littered throughout all interviews. The central characters in these stories were always family and friends. Perhaps embellished over the years, these stories were retold during gatherings” [42], p. 438. Furthermore, several studies suggested that friends share unique meanings, and leisure time with friends strengthens interpersonal relationships [42,43]. Based on this perspective, it can be presumed that time spent with friends strengthens satisfaction with interpersonal relationships and, consequently, life satisfaction.
(3) Time spent in nature. Outdoor leisure activities are preferred by users of different ages [28,39,44], genders [13], and cultural backgrounds [29,45,46]. Time spent in nature might encompass a wide range of leisure preferences, from leisure gardening, which evidenced contributing to life satisfaction of older adults [24], to outdoor activities, including enjoyment in walking or outdoor sports [34,35,47], or fishing and hunting [45,48,49]. Outdoor leisure preferences might reflect users’ connectedness to nature and environmental intelligence [49,50,51]. Based on previous research linking nature-based recreational activities and psychological wellbeing, it can be presumed that time spent in nature as a leisure preference significantly contributes to life satisfaction.
(4) Participation in events. Recreational activities such as participating in festivals or visiting museums, theaters, cinemas, or other events, are attracting the increased attention of researchers [12,18,52,53,54,55]. Research suggests that women are more likely than men to participate in highbrow leisure activities, but there is little evidence that highbrow leisure participation is related to family background or socioeconomic status [12]. Furthermore, research indicates that cultural participation is associated with personality trait openness to experience, as open users favor visiting art and historical museums and attending classical and pop concerts [54]. Participation in events, bringing recreational enjoyment and excitement, as a leisure preference, implies users’ openness and novelty-seeking, indicating possible links to life satisfaction.
(5) Watching television. There is a decades-long debate over the links between watching television (TV) and wellbeing. Some researchers established the benefits of TV watching, as, after a loss in the interpersonal sphere, television viewing can play a valuable role in adaptation processes [56]. Despite recognized temporal enjoyments in viewing, several studies pointed to its potential harms, including links to the “bedroom culture” phenomenon, lower physical activity, and self-control [6,25,44,57,58,59,60]. However, some research indicates that television as a leisure preference, especially for shared viewing with the family, can be a good tool for socializing and relaxation [7]. Consequently, it can also be linked to life satisfaction.
(6) Book reading. Book reading as a leisure activity plays a vital role in school-age years for lexical development [5], resulting in better verbal fluency, nonverbal problem-solving ability, and general knowledge [61]. There is also evidence on links between book reading and life quality in adulthood, as reading preferences expand a reader’s knowledge and reflect social differentiation [62]. Furthermore, research indicates that frequent readers achieve more and demonstrate higher IQ [63], and personality trait openness to experiences is a relevant indicator of reading preferences [54]. Based on previous research linking book reading with openness and numerous advantageous outcomes, it can be presumed that leisure book reading could also be linked to life satisfaction.

Even though many studies demonstrated links between leisure activities and life satisfaction, the question of which leisure preferences are most beneficial for psychological variables is still under-researched. Similarly, even though the links between personality traits, needs, and leisure preferences have been established, it is still under-researched which other psychological variables predict different leisure preferences. As a recent wave of positive psychology delineates the importance of mindfulness and psychological capital for life satisfaction, we considered it essential to analyze which leisure preferences contribute to mindfulness, psychological capital, and life satisfaction. In addition, we regarded it as important to analyze whether mindfulness, psychological capital, and life satisfaction predict different leisure preferences.

### 1.2. Mindfulness

The construct of mindfulness originated in Buddhism and Brahmanic traditions but is frequently used to refer to both a state or quality of mind and a form of meditation [64]. Kabat-Zinn defined mindfulness as the awareness that arises through paying purposeful attention to the present moment, which allows the nonjudgmental unfolding of experience moment by moment to “the way things are”, as in the Chinese notion of Tao [65]. 

Mindfulness has recently been extensively researched in various contexts, including its impact on improving academic performance [66], reducing anxiety symptoms [67] or stress [68], boosting immunity [9], improving heart rate variability or decreasing burnout [69], links to structural brain networks [70]. Mindfulness was also linked to social sustainability [71], work engagement [72], inhibiting malicious envy [73], or higher psychological capital [74]. Moreover, numerous studies reported links between mindfulness and psychological wellbeing [75,76]. However, the links between mindfulness and leisure preferences are under-researched. On the one hand, there is a question of which leisure activities can contribute to mindfulness as a state of mind. On the other hand, based on mindfulness research, it can be implied that mindfulness can contribute to satisfaction with various leisure activities, but the question of mindful leisure preferences and their links to life satisfaction needs a deeper analysis.

### 1.3. Psychological Capital

A concept of psychological capital (PsyCap) emerged along with the movement of positive psychology nearly two decades ago and received considerable attention from researchers [77,78,79,80,81,82,83,84,85,86,87,88]. Luthans et al. defined psychological capital as a multidimensional construct, reflecting four dimensions: self-efficacy, hope, resilience, and optimism [83]. Self–efficacy is considered as having the confidence to take on and put in the necessary effort to succeed at challenging tasks. Optimism is regarded as making positive attributions about succeeding now and in the future. Hope is considered as persevering toward goals and, when necessary, redirecting paths to goals to succeed. Resilience is defined as sustaining and bouncing back and beyond to attain success when beset by problems and adversity [83]. 

Numerous studies have documented associations between psychological capital (PsyCap) and life satisfaction [89,90] or psychological capital and quality of life [91]. Research indicated that persons who score higher on PsyCap are more likely to engage in opportunities to sustain and improve wellbeing and persist in achieving their goals [92].

Even though the concept of psychological capital was developed in an organizational context, based on research linking psychological capital to outcomes of general importance, researchers designed and validated a universal measure for the PsyCap construct, with applications for all domains of life [93]. It was established that psychological capital in different domains (relationship, health, work, and overall) is associated with positive affect, perceived social support, and life satisfaction. Furthermore, several studies revealed links between psychological capital and mindfulness [72,74]. 

However, the links between psychological capital and leisure preferences are still under-researched, even though leisure is one of the essential life domains. 

### 1.4. Life Satisfaction

The construct of life satisfaction indicates a cognitive evaluation of one’s life and is a component of subjective wellbeing or happiness [4,94,95]. Numerous studies have demonstrated links between leisure and happiness [21], leisure and fulfillment [32], and leisure and life satisfaction [96,97,98,99]. 

Newman et al. proposed five core psychological mechanisms that leisure potentially triggers to promote leisure subjective wellbeing: detachment-recovery, autonomy, mastery, meaning, and affiliation [4]. They argued that global subjective wellbeing is based on an evaluation of crucial life domains as leisure, work, and health, and leisure preferences might contribute to the various dimensions of subjective wellbeing in the leisure domain, which subsequently promotes global subjective wellbeing. A conceptual model proposed by Newman et al. on leisure and subjective wellbeing and psychological mechanisms as mediating factors delineate the importance of research on variables linked to leisure preferences and life satisfaction [4]. 

Furthermore, Sirgy et al., proposed a benefits theory of leisure wellbeing [11], which expanded Newman’s et al. model. In Sirgy et al.’s theory, twelve sets of mechanisms, reflecting basic and growth needs, impact satisfaction with leisure life and subjective wellbeing: leisure benefits related to safety, health, economic, hedonic, escape, sensation-seeking, symbolic, aesthetics, morality, mastery, relatedness, and distinctiveness. Sirgy et al. argued that the more a leisure activity delivers benefits related to basic and growth needs, the greater the likelihood that such an activity would contribute significantly to life satisfaction. On the other hand, it can be presumed that the more leisure activity contributes to life satisfaction, the greater is likelihood that particular leisure preference ensures the satisfaction of basic and growth needs.

This study did not focus on satisfaction or frustration of psychological needs in different leisure preferences, even though we admit the importance of such research. We considered it essential to explore links between leisure preferences and life satisfaction, mediated by earlier discussed mindfulness and psychological capital constructs, and to identify whether mindfulness, psychological capital, and life satisfaction predict different leisure preferences. Thus, this study aimed to identify associations between leisure preferences, mindfulness, psychological capital, and life satisfaction. Based on the previous research, we hypothesized that:

**Hypothesis** **1** **(H1).** *Respondents preferring different leisure types differ in life satisfaction, mindfulness, and psychological capital.*

**Hypothesis** **2** **(H2).** *Life satisfaction, mindfulness, and psychological capital are associated with different types of leisure.*

**Hypothesis** **3** **(H3).** *Associations between different leisure types and life satisfaction, mindfulness, and psychological capital differ between genders.*

**Hypothesis** **4** **(H4).** *Mindfulness and life satisfaction mediate links between leisure preferences and life satisfaction.*

**Hypothesis** **5** **(H5).** *Mindfulness, psychological capital, and life satisfaction are linked to different leisure preferences.*

## 2. Materials and Methods

### 2.1. Sample

This study was conducted in Lithuania and applied a cluster random sampling method. We have randomly selected two samples of private and public sector organizations from the Lithuanian organizations’ database. From within those samples, we have randomly selected a sample of respondents. Out of 1000 selected respondents, 601 respondents consented to participate in the study. However, the data of 15 participants were excluded from the further analysis due to not meeting the inclusion criteria.

The inclusion criteria for this research were: (1) 18 years old and older; (2) not unemployed or retired, but working, at least partially; (3) permanent residents of Lithuania. The exclusion criteria were the following: (1) younger than 18 years old; (2) just studying in educational institutions and not formally working (e.g., volunteers were excluded); (3) unemployed (candidates for a position were excluded); (4) fully retired; (5) not permanent residents of Lithuania.

In order to assess whether the data are not biased, the analysis of missing data has been performed. The data had no more than 20% of missing data of the variables. An omnibus statistical test for inspecting missing data confirmed that the data are missing completely at random. A test-by-test deletion was chosen for the analysis carried out with the SPSS. For the analysis carried out with the JASP, exclusion of cases pairwise/listwise and the full information maximum likelihood (FIML) methods were applied.

The study sample consisted of 586 participants, 104 males, 478 females, and four respondents indicated their gender as “other.” The sample of females was overrepresented in this study, but the sample sizes of two genders were sufficient to test the statistical hypothesis H3. The age of respondents ranged from 18 to 76 years (mean age was 42.06, SD = 13.29). In total, 47.5 percent of respondents were married, 34 percent were unmarried, 9 percent were divorced, and 4.2 percent were widowed. Fifty-five percent of respondents held Master’s degrees, 12 percent held Bachelor’s degrees, 24.2 percent finished Higher education, and 15 percent finished Secondary education. The possible respondents were asked to participate in the study online or during group meetings organized in several organizations and were informed that personal data (names, organization) are omitted in the questionnaire to ensure data confidentiality and anonymity. The research team received anonymous data of the sample. The procedure followed the General Data Protection Regulation (GDPR) guidelines and the Declaration of Helsinki and was approved by the Research Ethics Council at the Institute of Management and Psychology, Lithuania.

### 2.2. Instruments

#### 2.2.1. The SWLS

The authors measured respondents’ life satisfaction with the Satisfaction with Life Scale (SWLS), developed by E. Diener et al., a short 5-item instrument designed to assess global cognitive judgments of satisfaction with one’s life [100]. The response pattern followed a 7-point Likert scale ranging from totally disagree to agree strongly. Validation studies confirmed the one-dimensional structure of the SWLS, evidencing the instrument’s favorable psychometric properties [100,101,102,103]. 

#### 2.2.2. The PCQ-12

Psychological Capital or PsyCap was measured using the 12-item Psychological Capital Questionnaire (PCQ-12), applied to health, personal relationships, work, and overall life [92]. The PCQ-12 contains three items to measure efficacy, four items to measure hope, three to measure resilience, and two to measure optimism. The response pattern followed a 6-point Likert scale ranging from strongly disagree to agree strongly. Numerous studies demonstrated the reliability and validity of the PCQ-12 in various cultural contexts [104,105,106,107].

#### 2.2.3. The MAAS

Mindfulness was measured using the single-factor structure 15-item Mindful Attention Awareness Scale, developed by Brown and Ryan [108]. The items are rated on a 6-point Likert scale ranging from almost always to rarely. Respondents were given the following instruction: “below is a collection of statements about your everyday experience. Please answer according to what reflects your experience rather than what you think your experience should be”. The MAAS has been validated across multiple samples, evidencing good psychometric properties [75,109,110,111,112,113,114]. 

To ensure that the Lithuanian items corresponded as closely as possible to the English items, the original items of all instruments were translated into Lithuanian and back-translated. The McDonald’s ω and Cronbach’s α of the SWLS, the PCQ-12, and the MAAS in this study are displayed in Table 1.

#### 2.2.4. Leisure Preferences

Leisure preferences were evaluated, asking respondents a question: “How do you spend your free time?” and providing with the following list: “watching television”, “book reading”, “spending time in nature”, “spending time with family”, “spending time with friends”, “participating in events”, “going to meditation/yoga classes”, and “other”. The items were rated on a 4-point Likert scale ranging from not at all to very much. The option “meditation/yoga classes” was somewhat chosen by 3.8 percent of participants, and due to statistical insufficiency, we eliminated this variable from further analysis. The option “other” was somewhat chosen by 35 percent of participants, who indicated mostly activities like “gaming” or “social media” and somewhat “fitness” or “using SPA services.” However, this “other” option was also eliminated from further investigation, as the data were not sufficiently transparent and appropriate for statistical analyses.

### 2.3. Statistical Analyses

Statistical analyses were performed using SPSS v.26.0 (IBM Corp., Armonk, NY, USA) and JASP v. 0.16. (University of Amsterdam, Amsterdam, The Netherlands). Cronbach’s alphas and McDonald’s’ omegas were calculated to evaluate instruments’ reliability [115]. Confirmatory factor analysis (CFA) evaluated instruments’ validity [116]. A model fit evaluation was based on the GFI (Goodness of Fit Index), the CFI (Comparative Fit Index), the Normed Fit Index (NFI), the Tucker–Lewis coefficient (TLI), RMSEA (Root Mean Square Error of Approximation), and SRMR (Standardized Root Mean Square Residual), whereas the χ^2^ was used for descriptive purposes only [117]. The values higher than 0.90 for CFI, NFI, and TLI, and values lower than 0.08 for RMSEA and SRMR were considered indicative of a good fit, and *p*-values less than 0.05 were considered to be indicative of a good fit be statistically significant [118,119,120]. For descriptive purposes, bivariate nonparametric correlations between the study variables were analyzed using the Spearman correlation test [121]. Differences between groups were analyzed using Welch’s *t*-test [122]. Associations between the study variables were analyzed using multiple regression (forward method) and mediation analyses [123,124]. Data distribution was evaluated using the Shapiro-Wilk test, which showed the departure from normality. However, the distribution was moderately negatively skewed (Table 2).

## 3. Results

Based on responses regarding leisure preferences ranging from “not at all” to “very much,” we created conditional groups: if respondents checked answer “not at all” (0), they were considered to fall into the group of non-users of a particular leisure preference, and if respondents checked “somewhat” to “very much” (1–3), they fall into the group of particular leisure preference users. Frequencies of leisure preferences are displayed in Table 3.

In preliminary analyses, bivariate Spearman’s correlations between the types of leisure preferences and age were calculated (Table 4). Even though several significant correlations were identified, it is worth noting that this study revealed a significant negative correlation between age and spending time with friends (rho = −0.306, *p* < 0.001) or family (rho = −0.092, *p* < 0.001), evidencing the possible loneliness of elderly respondents.

The results of the confirmatory factor analysis are presented in Table 5. Model fit for all the instruments was good regarding GFI, CFI, TLI, NFI, and SRMR. However, the RMSEA for the PCQ-12 was higher the expected. One of the CFA’s main premises is that the residuals should not be correlated; thus, we present RMSEA of the PCQ-12 with uncorrelated residuals, even though RMSEA was considerably lower and demonstrated a good fit with correlated residuals. 

The descriptives and correlations between the PCQ-12, the MAAS, and the SWLS in this study are displayed in Table 6.

Table 7 displays Spearman’s correlations between types of leisure preferences and the PCQ-12, the MAAS, and the SWLS.

In the main analysis, we conducted several independent samples *t*-tests to test H1, which presumed that respondents preferring different leisure types differ in life satisfaction, mindfulness, and psychological capital. Firstly, we compared respondents who prefer watching television with those who reported not spending free time on TV. The results show (Table 8) that respondents who did not spend free time on TV demonstrated significantly higher scores on life satisfaction, mindfulness, and psychological capital, including PsyCap overall, PsyCap work, PsyCap relationship, and PsyCap health.

The comparison of respondents who prefer and do not prefer attending events (Table 9) showed that those who prefer attending events reported significantly higher scores on life satisfaction, PsyCap overall, and PsyCap work. 

The comparative analysis of those who prefer and do not prefer spending leisure time in nature (Table 10) indicated that respondents who prefer nature also score significantly higher on psychological capital, including PsyCap overall, PsyCap relationship, and PsyCap health.

Most importantly, the comparison of respondents who prefer and do not prefer spending time with family (Table 11) showed that those who chose the family as a leisure preference also demonstrated significantly higher scores on life satisfaction, mindfulness, and psychological capital, including PsyCap overall, PsyCap work, PsyCap relationship, and PsyCap health.

Surprisingly, no significant differences were found in any of the study variables in groups of respondents who prefer or do not prefer book reading or spending time with friends. 

To sum up, Welch’s *t*-test analysis partially confirmed H1, evidencing some differences in life satisfaction, mindfulness, and psychological capital, including PsyCap overall, PsyCap work, PsyCap relationship, and PsyCap health in groups of participants with different leisure preferences. 

Furthermore, to test H2, assuming that different types of leisure associate with life satisfaction, mindfulness, and psychological capital, we conducted multiple linear regression (forward method) analyses in the total sample. The results of multiple regression models, when the dependent variables are life satisfaction, mindfulness, and psychological capital, and the predictors are leisure preferences, are displayed in Table 12.

Multiple regression analyses showed several significant regression equations concerning the dependent variable of life satisfaction. In model 1, life satisfaction was significantly predicted by time spent with family. In model 2, significant predictors of life satisfaction were time spent with family and participating in events. Model 3 indicated that life satisfaction was significantly predicted by time spent with family, participating in events, and the absence of watching television. Next, regression analysis showed a significant regression equation concerning the dependent variable of mindfulness. Predicted mindfulness equalled 4.294–0.045 (watching television) points. In other words, mindfulness was predicted by not watching television in this model. Next, several significant regression equations were found regarding the dependent variable of psychological capital. Model 1 indicated that the absence of watching television significantly predicted psychological capital. In model 2, psychological capital was predicted by time spent with family and not watching television. In model 3, significant predictors of psychological capital were spending time with family, participating in events, and not watching television. Model 4 indicated that not watching television but spending time with family, participating in events, and spending time in nature significantly predicts psychological capital.

Thus, multiple regression analyses partially confirmed H2, evidencing how different types of leisure are associated with life satisfaction, mindfulness, and psychological capital. 

Furthermore, to test H3, presuming that associations between different leisure types and life satisfaction, mindfulness, and psychological capital differ between genders, we conducted multiple linear regression (forward method) analyses in two gender groups separately. The results of multiple regression models in groups of females and males, when the dependent variables are life satisfaction, mindfulness, and psychological capital, and the predictors are leisure preferences, are displayed in Table 13.

Multiple regression analyses in both genders showed several significant regression equations concerning the dependent variable of life satisfaction. In a group of males, life satisfaction was significantly predicted by time spent in nature. In a group of females, model 1, a significant predictor of life satisfaction was time spent with family, and model 2 indicated that significant predictors of females’ life satisfaction were spending time with family and participating in events. Next, regression analysis showed several significant regression equations concerning the dependent variable of mindfulness. In a group of males, mindfulness was significantly predicted by book reading. In a group of females, mindfulness was predicted by not watching television. Next, several significant regression equations were found regarding the dependent variable of psychological capital. In a group of males, model 1, overall psychological capital was significantly predicted by spending time with family. Model 2 indicated that spending time with family and book reading significantly predicted the psychological capital of males. In a group of females, model 1, overall psychological capital was predicted by not watching television. In model 2, significant predictors of females’ psychological capital were participating in events and not watching television. In model 3, females’ psychological capital was significantly predicted by participating in events, not watching television, and spending time with family. Model 4 indicated that females’ psychological capital was significantly predicted by not watching television but spending time with family, participating in events, and spending time in nature.

Therefore, multiple regression analyses partially confirmed H3, evidencing that associations between different leisure types and life satisfaction, mindfulness, and psychological capital differ between genders.

Furthermore, to test H4, assuming that mindfulness and psychological capital mediate links between leisure preferences and life satisfaction, we initially performed multiple mediation analyses based on the conceptual model of links between leisure preferences, psychological capital, mindfulness, and life satisfaction. However, most of the links were statistically insignificant. For clarity, we present separately two significant models demonstrating the role of mindfulness and the role of psychological capital as mediators.

**Figure 1:**
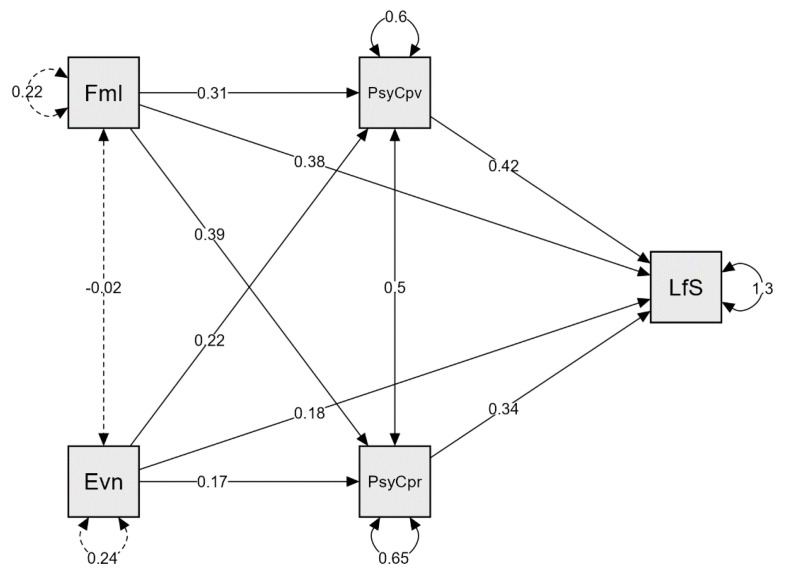
Mediation analysis: path plot in the total sample, the role of psychological capital as a mediator. Fml: spending time with family; Evn: participating in events; PsyCpv: PsyCap overall; PsyCpr: PsyCap relationship; LfS: life satisfaction.

In a model shown in Figure 1, the outcome variable for the mediation analysis was life satisfaction. Based on previous analyses, the predictor variables were spending time with family and participation in events, and the mediator variables were the PsyCap overall and PsyCap relationship. 

The mediation analysis results indicating the role of psychological capital are presented in Table 14. The indirect effects of spending time with family and participating in events on life satisfaction were statistically significant. Spending time with family significantly predicted psychological capital overall and psychological capital relationship, which significantly predicted life satisfaction. Participating in events significantly predicted overall psychological capital, subsequently predicting life satisfaction. However, R² for life satisfaction in the total sample was 0.254, PsyCap overall R2 was 0.049, and PsyCap relationship R2 was 0.055.

**Figure 2:**
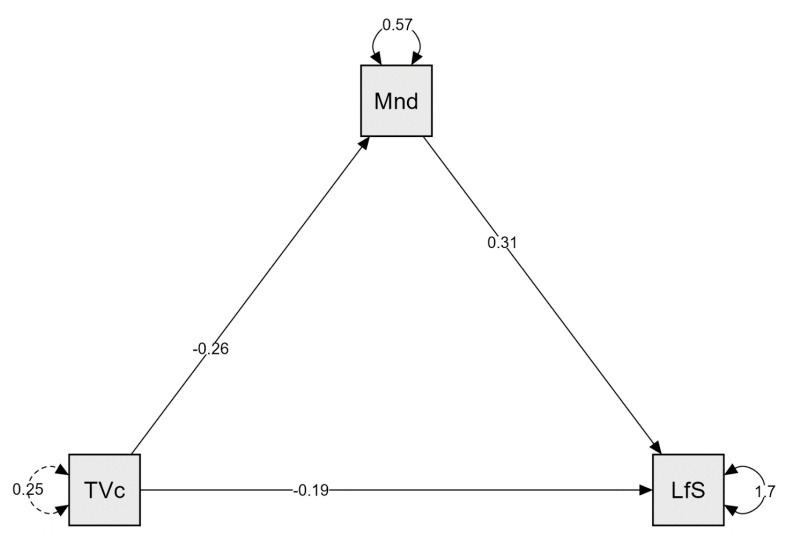
Mediation analysis: path plot in the total sample, the role of mindfulness as a mediator. TVc: watching television; Mnd: mindfulness; LfS: life satisfaction.

In a model shown in Figure 2, the outcome variable for the mediation analysis was life satisfaction, the predictor variable was watching television, and the mediator variable was mindfulness. 

The mediation analysis results indicating the role of mindfulness are presented in Table 15. The indirect effect of watching television on life satisfaction was statistically significant, even though R² for life satisfaction was 0.043, and R² for mindfulness was 0.028.

Thus, mediation analyses partially confirmed H4, evidencing that mindfulness and psychological capital mediate links between leisure preferences and life satisfaction. Mindfulness mediated the link between watching television and life satisfaction, and psychological capital mediated links between spending time with family, participating in events, and life satisfaction.

Next, to test H5, assuming that mindfulness, psychological capital, and life satisfaction are linked to different leisure preferences, multiple regression analyses (forward method) were conducted (Table 16).

Regression analysis showed a significant regression equation concerning the dependent variable of spending time with family. Life satisfaction was a significant predictor of spending time with family as a leisure preference F (1, 259) = 16.510, *p* < 0.001, with R2 = 0.060. Predicted spending time with family was equal to 2.193 + 0.469 (life satisfaction) points. Spending time with family increased + 0.469 for each life satisfaction point (*p* < 0.001). Thus, life satisfaction contributed significantly to the model and was a significant predictor of spending time with family as a leisure preference. Next, predicted watching television was equal to 5.095–0.622 (PsyCap relationship) points, F (1, 258) = 8.428, *p* = 0.004, with R2 = 0.032. Watching television decreased—0.622 points for each PsyCap relationship point. Thus, the PsyCap relationship was a significant predictor of watching television. Then, predicted participation in events increased +0.480 points for each PsyCap overall point, F (1, 259) = 5.066, *p* = 0.025, with R2 = 0.019. However, mindfulness was not a significant predictor of any leisure preferences. 

Thus, regression analyses partially confirmed H5, evidencing links between leisure preferences and life satisfaction and psychological capital. Life satisfaction was associated with spending time with family, PsyCap relationship was associated with spending less time on TV, and PsyCap was overall associated with participation in events. However, these findings need further investigation.

## 4. Discussion

This study was a preliminary attempt to explore links between leisure preferences and mindfulness, psychological capital, and life satisfaction. It explored which leisure preferences contribute to mindfulness, psychological capital, and life satisfaction. In addition, it aimed to assess whether mindfulness, psychological capital, and life satisfaction are linked to different leisure preferences. Many previous studies established links between leisure preferences and life satisfaction [23,31], links between life satisfaction and psychological capital [89,90,125,126,127,128], life satisfaction and mindfulness [129,130], or mindfulness and psychological capital [72,74,76,131]. Previous studies also examined links between several types of leisure activities and psychological capital [99,132] or links between some activities (meditation, physical activity) and mindfulness [9,133,134,135]. However, the links between leisure preferences, mindfulness, psychological capital, and life satisfaction have not previously been investigated in the same study. 

The context of restrictions due to COVID-19 makes the findings of this study important as this research draws attention to possible psychological benefits of different daily leisure preferences. In addition, it proposes some insights on psychological antecedents of leisure choices, as established by previous research [2]. 

The research was focused on three psychological constructs: mindfulness, proposed by Kabat-Zinn [65]; life satisfaction, proposed by Diener et al. [94]; and psychological capital, proposed by Luthans et al. [136].

In this study, we hypothesized (H1) that respondents preferring different leisure types differ in life satisfaction, mindfulness, and psychological capital. The findings partially confirmed H1, evidencing some differences in life satisfaction, mindfulness, and psychological capital, including PsyCap overall, PsyCap work, PsyCap relationship, and PsyCap health in groups of participants with different leisure preferences. The results show that respondents who did not spend free time watching television demonstrated significantly higher scores on life satisfaction, mindfulness, and psychological capital, including PsyCap overall, PsyCap work, PsyCap relationship, and PsyCap health. Next, the findings indicat that those who prefer attending events score higher on life satisfaction, PsyCap overall, and PsyCap work. Most importantly, the results show that those who spend time with family as a leisure preference demonstrated significantly higher scores on life satisfaction, mindfulness, and psychological capital, including PsyCap overall, PsyCap work, PsyCap relationship, and PsyCap health. Surprisingly, no significant differences were found in any of the study variables in groups of respondents who prefer or do not prefer book reading or spending time with friends. These results might be partially linked to previous research suggesting associations between life satisfaction and leisure preferences, namely, family [12,39,40,55,137,138], watching television [5,7,10,56,60,139,140,141,142], or participation in events [54,58,143]. However, it is unclear why the differences concerning book reading or spending time with friends were not significant in this study and why those who prefer spending leisure time with a family score higher on mindfulness and psychological capital.

Furthermore, in this study, we hypothesized (H2) that different types of leisure preferences link to life satisfaction, mindfulness, and psychological capital. The findings reveal that time spent with family significantly predicts life satisfaction. In another model, life satisfaction was also significantly predicted by time spent with family, participating in events, and the absence of watching television. Moreover, mindfulness was also predicted by not watching television. Next, the results demonstrate that not watching television but spending time with family, participating in events, and spending time in nature significantly predicts psychological capital. Thus, the findings partially confirmed H2, evidencing how different types of leisure predict life satisfaction, mindfulness, and psychological capital. These findings align with some prior research on links between life satisfaction and spending time with family [41], watching television [54], and participation in events [14,44,143,144]. However, the question of why watching television decreases mindfulness and psychological capital and why spending time in nature or with friends or book reading is not significant for life satisfaction needs further investigation and warns that these results should be regarded with caution. 

This study also hypothesized (H3) that associations between different leisure types and life satisfaction, mindfulness, and psychological capital differ between genders, as previous research indicated possible effects [12,13]. The findings revealed that males’ life satisfaction was significantly predicted by time spent in nature, while females’ satisfaction was predicted by spending time with family and participating in events. Next, males’ mindfulness was significantly predicted by book reading, and females’ mindfulness was predicted by not watching television. Then, the psychological capital of males was significantly predicted by spending time with family and book reading, and females’ psychological capital was predicted by not watching television but spending time with family, participating in events, and spending time in nature. Therefore, the findings partially confirmed H3, showing that associations between leisure types and life satisfaction, mindfulness, and psychological capital differ between genders. These results are consistent with some previous studies suggesting the possible effect of gender on leisure preferences and the related variables [96,144,145]. However, the mechanism underlying the links’ specifics in different genders is still unclear and needs further investigation. 

Furthermore, this research assumed (H4) that mindfulness and psychological capital mediate links between leisure preferences and life satisfaction. The findings show that mindfulness mediated the link between watching television and life satisfaction, and psychological capital mediated links between spending time with family, participating in events, and life satisfaction. Mediation analyses revealed that the indirect effects of spending time with family and participating in events on life satisfaction were statistically significant: spending time with family significantly predicted psychological capital overall and psychological capital relationship, which significantly predicted life satisfaction. Participating in events significantly predicted overall psychological capital, subsequently predicting life satisfaction. Moreover, the indirect effect of watching television on life satisfaction was also statistically significant. Thus, H4 was partially confirmed, evidencing that mindfulness and psychological capital mediate links between leisure preferences and life satisfaction. These results signify that leisure preferences and life satisfaction are interrelated constructs mediated by psychological variables, as indicated by previous studies [17,21,28,45,132,137,141,143,146,147,148,149,150]. However, these associations’ details concerning particular leisure preferences require a more grounded approach, preferably including personality traits as predictors [96] and psychological needs satisfaction [134] as covariates in further research.

This study also hypothesized (H5) that mindfulness, psychological capital, and life satisfaction could be linked to different leisure preferences. The findings demonstrate that life satisfaction was a significant predictor of spending time with family as a leisure preference. Next, the PsyCap relationship was a significant predictor of (not) watching television, and PsyCap overall was a significant predictor of participation in events. However, contrary to the expected, mindfulness was not a significant predictor of any leisure preferences in this study. Even though the results partially confirm H5, as life satisfaction is associated with spending time with family, the PsyCap relationship is associated with spending less time watching television, and PsyCap overall is associated with participation in events, which might be partially explained by previous research [11,19,23,31,96] these findings need further investigation.

However, we acknowledge that the associations found in the current study may change depending on the contextual factors in which the leisure activities are chosen. Recent studies indicated that the associations between leisure activities and well-being could be moderated by the timing, duration, types of content, and social factors of the leisure activities. E.g., Hartanto et al. (2021) indicated the moderating role of contextual factors in the associations between video gaming and well-being [151]; Tooth et al. (2021) delineated the effect of family context related to screen time, child behavior, and health-related quality of life [152]. These findings impose the need for detailed exploration of contextual factors in future research.

## 5. Limitations

This study is a preliminary attempt to explore links between leisure preferences and psychological variables of mindfulness, psychological capital, and life satisfaction, and it has many limitations. The first limitation is the sample size and sample characteristics. The research sample was not representative; females were overrepresented, and the samples were statistically insufficient for structural equation modeling (SEM) regarding comparing SEMs in different groups. Thus, the results should be cautioned due to the sample characteristics. The second limitation is cultural context. The study was conducted in Lithuania, which since 2004 has belonged to the European Union but is a post-soviet country. Possibly, the same research in other world regions would generate different results. Comparison of previous studies suggests that leisure research is sensitive to cultural factors [150,151,152,153]. Thus, the findings should be taken with concern. Next, the measurement of leisure preferences was based on the authors’ created instrument, which intended to assess the prevalence of certain leisure preferences but did not measure leisure satisfaction. Besides, this instrument did not include leisure options like “social media”, “gaming”, “SPA services”, or many others, which are “the must” to be included in future research. Besides, recent studies have suggested analyzing “screen-based” recreational activities [147,154,155,156]. Next, in future research, it will be important to adjust for demographic variables such as age to ensure that the results were not confounded. Finally, the results indicate a necessity for longitudinal or experimental research design because it is possible only to identify significant relationships among the examined variables based on the data obtained. This research can be used to describe characteristics that exist in the selected sample, but not to determine cause-and-effect relationships between different variables.

## 6. Conclusions

This study provided some evidence that (H1) respondents preferring different leisure types differ in life satisfaction, mindfulness, and psychological capital; (H2) life satisfaction, mindfulness, and psychological capital are associated with different types of leisure; (H3) associations between different leisure types and life satisfaction, mindfulness, and psychological capital partially differ between genders. The findings also reveal that (H4) mindfulness and life satisfaction mediate links between leisure preferences and life satisfaction, and (H5) mindfulness, psychological capital, and life satisfaction are linked to different leisure preferences. The results show that respondents who did not spend free time watching television scored higher on life satisfaction, mindfulness, and psychological capital. Participants who preferred attending events scored higher on life satisfaction and psychological capital. Participants who preferred spending time with family as a leisure preference scored significantly higher on life satisfaction, mindfulness, and psychological capital, including PsyCap overall, PsyCap work, PsyCap relationship, and PsyCap health. The findings also reveal that time spent with family is significantly associated with life satisfaction. Besides, males’ life satisfaction was significantly associated with time spent in nature, while females’ satisfaction was associated with spending time with family and participating in events. Males’ mindfulness was significantly associated with book reading, and females’ mindfulness was associated with not watching television. Males’ psychological capital was significantly associated with spending time with family and book reading, and females’ psychological capital was associated with not watching television but spending time with family, participating in events, and spending time in nature. The findings also show that mindfulness mediated the link between watching television and life satisfaction, and psychological capital mediated links between spending time with family, participating in events, and life satisfaction. The findings demonstrated that life satisfaction is also significantly associated with spending time with family as a leisure preference. This study also evidenced a significant negative association between age and spending time with friends or family, evidencing the possible loneliness of elderly respondents. However, due to limitations of this study, including sample size and characteristics, cultural context, and research design, the research findings would preferably be regarded thoughtfully.

## Figures and Tables

**Table 1:** Cronbach alphas and McDonald’s omegas for the SWLS, the PCQ-12, and the MAAS.

Scales	McDonald’s ω	Cronbach’s α
Psychological Capital, overall	0.890	0.889
Psychological Capital, work	0.893	0.892
Psychological Capital, relationship	0.891	0.890
Psychological Capital, health	0.884	0.882
Mindful Attention Awareness	0.862	0.859
Satisfaction with Life	0.882	0.881

**Table 2:** The data distribution for the SWLS, the PCQ-12, and the MAAS.

Scales	Shapiro-Wilk	*p*	Skewness	SE	Kurtosis	SE
Psychological Capital, overall	0.987	>0.001	−0.352	0.117	0.318	0.234
Psychological Capital, work	0.991	0.008	−0.254	0.117	−0.114	0.234
Psychological Capital, relationship	0.986	>0.001	−0.368	0.118	0.024	0.235
Psychological Capital, health	0.990	0.004	−0.318	0.118	0.234	0.235
Mindful Attention Awareness	0.984	0.001	−0.465	0.134	0.286	0.267
Satisfaction with Life	0.987	>0.001	−0.122	0.103	−0.548	0.206

**Table 3:** Frequencies of leisure preferences.

Types of Leisure Preferences	Total, %	Total, *n*	Female, *n*	Male, *n*
Spending time in nature	51.87	305	249	52
Spending time with family	67.57	398	326	68
Spending time with friends	40.23	237	183	51
Reading books	59.35	349	311	35
Watching TV	44.14	260	205	51
Participating in events	38.71	228	197	27

**Table 4:** Bivariate Spearman’s correlations between the types of leisure preferences and age.

Types of Leisure Preferences	Nature	Family	Friends	Books	TV	Age
Spending time in nature	-					0.017
Spending time with family	0.034	-				−0.092 *
Spending time with friends	0.069	−0.048	-			−0.306 **
Reading books	0.018	−0.129 **	0.008	-		0.239 **
Watching TV	0.016	−0.038	0.083 *	0.224 ***	−	0.216 **
Participating in events	0.036	−0.059	0.153 ***	0.240 ***	0.103 *	0.117 **

* *p* < 0.05, ** *p* < 0.01, *** *p* < 0.001.

**Table 5:** Confirmatory factor analysis (CFA): Model fit indices for the PCQ-12, the MAAS, and the SWLS.

Factor Model	χ^2^	Df	GFI	CFI	TLI	NFI	RMSEA [90% CI]	SRMR
Psychological capital, overall	320.848	44	0.985	0.978	0.972	0.974	0.120 [0.108–0.133]	0.067
Psychological capital, work	255.238	54	0.989	0.986	0.983	0.983	0.093 [0.082–0.105]	0.056
Psychological capital, relationship	124.316	35	0.990	0.987	0.984	0.984	0.091 [0.078–0.104]	0.054
Psychological capital, health	506.283	54	0.975	0.964	0.956	0.960	0.140 [0.129–0.151]	0.083
Mindful Attention Awareness	156.282	90	0.991	0.993	0.991	0.983	0.047 [0.034–0.059]	0.051
Satisfaction with Life	6406.246	5	0.999	0.999	0.999	0.999	0.022 [0.000–0.066]	0.014

**Table 6:** The means, standard deviations, and bivariate correlations between the PCQ-12, the MAAS, and the SWLS.

Scales	Mean	SD	1	2	3	4	5
1. Psychological Capital, overall	4.406	0.792	-				
2. Psychological Capital, work	4.398	0.779	0.786 ***	-			
3. Psychological Capital, relationship	4.339	0.834	0.802 ***	0.689 ***	-		
4. Psychological Capital, health	4.220	0.809	0.729 ***	0.666 ***	0.721 ***	-	
5. Mindful Attention Awareness	4.189	0.766	0.321 ***	0.270 ***	0.348 ***	0.323 ***	-
6. Satisfaction with Life	4.276	1.315	0.469 ***	0.387 ***	0.470 ***	0.422 ***	0.199 ***

*** *p* < 0.001.

**Table 7:** Bivariate correlations between types of leisure preferences and the PCQ-12, the MAAS, and the SWLS.

Scales	Nature	Family	Friends	Books	TV	Events
1. Psychological Capital, overall	0.100 *	0.182 ***	0.026	−0.038	−0.189 ***	0.107 *
2. Psychological Capital, work	0.090	0.173 ***	−0.057	0.019	−0.090	0.117 *
3. Psychological Capital, relationship	0.106 *	0.239 ***	0.006	−0.077	−0.158 **	0.063
4. Psychological Capital, health	0.118 *	0.133 **	−0.003	−0.051	−0.140 **	0.056
5. Mindful Attention Awareness	0.027	0.064	−0.026	−0.027	−0.147 **	0.013
6. Satisfaction with Life	0.014	0.185 ***	0.059	−0.007	−0.081	0.108 *

* *p* < 0.05, ** *p* < 0.01, *** *p* < 0.001.

**Table 8:** Comparison (Welch’s *t*-test) of life satisfaction, mindfulness, and psychological capital in groups of respondents who prefer and do not prefer TV.

Variables	Leisure Types	Mean	SD	*t*	df	*p*	Mean Difference	SE Difference	Cohen’s d
Life satisfaction	No TV	4.393	1.245	2.367	484.597	0.018	0.269	0.114	0.204
	TV	4.124	1.393						
Mindfulness	No TV	4.303	0.724	3.115	294.607	0.002	0.264	0.085	0.348
	TV	4.038	0.795						
PsyCap overall	No TV	4.545	0.751	4.187	375.429	<0.001	0.322	0.077	0.411
	TV	4.223	0.817						
PsyCap work	No TV	4.466	0.761	2.042	379.902	0.042	0.157	0.077	0.201
	TV	4.309	0.805						
PsyCap relationship	No TV	4.459	0.786	3.334	366.978	<0.001	0.272	0.082	0.329
	TV	4.187	0.869						
PsyCap health	No TV	4.334	0.820	3.430	401.235	<0.001	0.267	0.078	0.335
	TV	4.067	0.774						

**Table 9:** Comparison (Welch’s *t*-test) of life satisfaction, mindfulness, and psychological capital in groups of respondents who prefer and do not prefer participation in events.

Variables	Leisure Types	Mean	SD	*t*	df	*p*	Mean Difference	SE Difference	Cohen’s d
Life satisfaction	Not Events	4.171	1.311	−2.443	443.701	0.015	−0.280	0.114	−0.213
	Events	4.450	1.311						
Mindfulness	Not Events	4.176	0.741	−0.303	230.294	0.762	−0.027	0.090	−0.035
	Events	4.203	0.810						
PsyCap overall	Not Events	4.337	0.801	−2.397	346.422	0.017	−0.187	0.078	−0.238
	Events	4.524	0.773						
PsyCap work	Not Events	4.323	0.789	−2.620	348.586	0.009	−0.201	0.077	−0.260
	Events	4.524	0.757						
PsyCap relationship	Not Events	4.292	0.849	−1.634	356.069	0.103	−0.133	0.082	−0.162
	Events	4.426	0.800						
PsyCap health	Not Events	4.172	0.834	−1.596	360.574	0.111	−0.126	0.079	−0.158
	Events	4.299	0.765						

**Table 10:** Comparison (Welch’s *t*-test) of life satisfaction, mindfulness, and psychological capital in groups of respondents who prefer and do not prefer spending leisure time in nature.

Variables	Leisure Types	Mean	SD	*t*	df	*p*	Mean Difference	SE Difference	Cohen’s d
Life satisfaction	Not nature	4.254	1.336	−0.350	549.266	0.727	−0.039	0.112	−0.030
	Nature	4.293	1.299						
Mindfulness	Not nature	4.131	0.809	−1.140	291.378	0.255	−0.098	0.086	−0.128
	Nature	4.229	0.732						
PsyCap overall	Not nature	4.297	0.799	−2.669	415.595	0.008	−0.205	0.077	−0.259
	Nature	4.501	0.783						
PsyCap work	Not nature	4.327	0.792	−1.765	412.711	0.078	−0.134	0.076	−0.172
	Nature	4.461	0.773						
PsyCap relationship	Not nature	4.236	0.832	−2.445	413.661	0.015	−0.197	0.081	−0.238
	Nature	4.433	0.826						
PsyCap health	Not nature	4.101	0.789	−2.827	417.945	0.005	−0.221	0.078	−0.275
	Nature	4.321	0.818						

**Table 11:** Comparison (Welch’s *t*-test) of life satisfaction, mindfulness, and psychological capital in groups of respondents who prefer and do not prefer spending leisure time with family.

Variables	Leisure Types	Mean	SD	*t*	df	*p*	Mean Difference	SE Difference	Cohen’s d
Life satisfaction	Not family	3.851	1.381	−5.154	314.770	<0.001	−0.626	0.121	−0.477
	Family	4.477	1.237						
Mindfulness	Not family	4.107	0.795	−1.111	150.462	0.268	−0.108	0.097	−0.140
	Family	4.215	0.755						
PsyCap overall	Not family	4.193	0.846	−3.647	232.232	<0.001	−0.311	0.085	−0.388
	Family	4.504	0.753						
PsyCap work	Not family	4.200	0.783	−3.518	244.616	<0.001	−0.287	0.082	−0.371
	Family	4.487	0.768						
PsyCap relationship	Not family	4.064	0.843	−4.632	237.296	<0.001	−0.403	0.087	−0.491
	Family	4.467	0.798						
PsyCap health	Not family	4.076	0.908	−2.311	215.592	0.022	−0.209	0.090	−0.250
	Family	4.285	0.755						

**Table 12:** Multiple regression model, the dependent variables are life satisfaction, mindfulness, and psychological capital, and the predictors are leisure preferences.

Dependent Variable	Predictors/ Models	Unstandardized Coefficients	Standardized Coefficients		*t*	Sig.	R	R2	Adjusted R2	F	Sig.
		B	Std. Error	Beta							
Life Satisfaction	1 (Constant)	3.862	0.096		40.344	0.000	0.217	0.047	0.045	27.433 (1,555)	<0.001
	Spend time with family	0.104	0.020	0.217	5.238	0.000					
	2 (Constant)	3.719	0.107		34.802	0.000	0.249	0.062	0.058	18.205 (2,555)	<0.001
	Spend time with family	0.110	0.020	0.229	5.537	0.000					
	Participate in events	0.062	0.021	0.121	2.933	0.003					
	3 (Constant)	3.819	0.116		32.814	0.000	0.263	0.069	0.064	13.728 (3,555)	<0.001
	Spend time with family	0.108	0.020	0.225	5.458	0.000					
	Participate in events	0.065	0.021	0.127	3.071	0.002					
	Watch TV	−0.043	0.020	−0.088	−2.131	0.034					
Mindfulness	1 (Constant)	4.294	0.055		77.383	0.000	0.162	0.026	0.023	8.775 (1,327)	0.003
	Watch TV	−0.045	0.015	−0.162	−2.962	0.003					
PsyCap overall	1 (Constant)	4.534	0.049		91.718	0.000	0.190	0.036	0.034	16.072 (1,428)	<0.001
	Watch TV	−0.057	0.014	−0.190	−4.009	0.000					
	2 (Constant)	4.323	0.073		59.228	0.000	0.263	0.069	0.065	15.878 (2,428)	<0.001
	Watch TV	−0.057	0.014	−0.192	−4.102	0.000					
	Spend time with family	0.053	0.014	0.182	3.892	0.000					
	3 (Constant)	4.236	0.079		53.668	0.000	0.293	0.086	0.079	13.309 (3,428)	<0.001
	Watch TV	−0.058	0.014	−0.195	−4.203	0.000					
	Spend time with family	0.056	0.014	0.193	4.141	0.000					
	Participate in events	0.041	0.015	0.129	2.770	0.006					
	4 (Constant)	4.132	0.087		47.343	0.000	0.318	0.101	0.093	11.932 (4,428)	<0.001
	Watch TV	−0.058	0.014	−0.195	−4.235	0.000					
	Spend time with family	0.055	0.013	0.189	4.083	0.000					
	Participate in events	0.043	0.015	0.137	2.955	0.003					
	Spend time in nature	0.037	0.014	0.124	2.686	0.008					

**Table 13:** Multiple regression model, the dependent variables are life satisfaction, mindfulness, and psychological capital, and the predictors are leisure preferences.

Dependent Variable	Predictors/ Models	Unstandardized Coefficients	Standardized Coefficients		*t*	Sig.	R	R2	Adjusted R2	F	Sig.
		B	Std. Error	Beta							
Life Satisfaction	Males										
	1 (Constant)	4.061	0.194		20.981	0.000	0.209	0.044	0.034	4.336 (1,96)	0.040
	Spend time in nature	0.106	0.051	0.209	2.082	0.040					
	Females										
	1 (Constant)	3.834	0.105		36.389	0.000	0.230	0.053	0.051	25.229 (1,451)	0.000
	Spend time with family	0.109	0.022	0.230	5.023	0.000					
	2 (Constant)	3.663	0.118		31.082	0.000	0.271	0.073	0.069	17.736 (2,451)	0.000
	Spend time with family	0.115	0.022	0.243	5.331	0.000					
	Participate in events	0.072	0.023	0.142	3.123	0.002					
Mindfulness	Males										
	1 (Constant)	3.802	0.145		26.203	0.000	0.330	0.109	0.093	6.722 (1,56)	0.012
	Read books	0.107	0.041	0.330	2.593	0.012					
	Females										
	1 (Constant)	4.315	0.058		74.337	0.000	0.154	0.024	0.020	6.397 (1,265)	0.012
	Watch TV	−0.042	0.016	−0.154	−2.529	0.012					
PsyCap overall	Males										
	1 (Constant)	4.101	0.190		21.539	0.000	0.265	0.070	0.058	5.902 (1,79)	0.017
	Spend time with family	0.096	0.039	0.265	2.429	0.017					
	2 (Constant)	3.870	0.214		18.090	0.000	0.353	0.124	0.102	5.466 (2,79)	0.006
	Spend time with family	0.115	0.039	0.319	2.912	0.005					
	Read books	0.088	0.040	0.238	2.179	0.032					
	Females										
	1 (Constant)	4.525	0.051		88.150	0.000	0.212	0.045	0.042	16.099 (1,343)	0.000
	Watch TV	−0.059	0.015	−0.212	−4.012	0.000					
	2 (Constant)	4.440	0.059		75.542	0.000					
	Watch TV	−0.061	0.015	−0.217	−4.148	0.000	0.260	0.068	0.062	12.401 (2,343)	0.000
	Participate in events	0.044	0.015	0.151	2.891	0.004					
	3 (Constant)	4.254	0.083		51.249	0.000	0.306	0.094	0.086	11.731 (3,343)	0.000
	Watch TV	−0.060	0.014	−0.214	−4.144	0.000					
	Participate in events	0.048	0.015	0.163	3.139	0.002					
	Spend time with family	0.044	0.014	0.162	3.123	0.002					
	4 (Constant)	4.161	0.093		44.811	0.000	0.326	0.106	0.096	10.087 (4,343)	0.000
	Watch TV	−0.060	0.014	−0.213	−4.147	0.000					
	Participate in events	0.050	0.015	0.170	3.299	0.001					
	Spend time with family	0.044	0.014	0.160	3.109	0.002					
	Spend time in nature	0.031	0.014	0.112	2.183	0.030					

**Table 14:** Mediation analysis results in the total sample of respondents: the role of psychological capital as a mediator.

Paths			Coeff.	Std. Error	z-Value	*p*	95% CI Lower Upper	
Direct effects								
Family →	Life Satisfaction		0.375	0.110	3.405	<0.001	0.159	0.592
Events →	Life Satisfaction		0.180	0.104	1.734	0.083	0.024	0.384
Indirect effects								
Family →	PsyCap overall →	Life Satisfaction	0.132	0.050	2.646	0.008	0.034	0.230
Family →	PsyCap relationship →	Life Satisfaction	0.134	0.053	2.542	0.011	0.031	0.237
Events →	PsyCap overall →	Life Satisfaction	0.093	0.041	2.244	0.025	0.012	0.174
Events →	PsyCap relationship →	Life Satisfaction	0.058	0.033	1.736	0.083	0.007	0.123
Total effects								
Family →	Life Satisfaction		0.642	0.116	5.544	<0.001	0.415	0.869
Events →	Life Satisfaction		0.331	0.111	2.980	0.003	0.113	0.549
Total indirect effects								
Family →	Life Satisfaction		0.266	0.064	4.191	<0.001	0.142	0.391
Events →	Life Satisfaction		0.151	0.058	2.605	0.009	0.037	0.265
Residual covariances								
PsyCap overall <-> PsyCap relationship			0.501	0.038	13.111	0.000	0.426	0.576
Life satisfaction R2 = 0.254; PsyCap overall R2 = 0.049; PsyCap relationship R2 = 0.055.								

Note. Delta method standard errors, normal theory confidence intervals, ML estimator.

**Table 15:** Mediation analysis results in the total sample of respondents: the role of mindfulness as a mediator.

Paths			Coeff.	Std. Error	z-Value	*p*	95% CI	
							Lower	Upper
Direct effects								
TV →	Life Satisfaction		−0.190	0.114	−1.671	0.095	−0.413	−0.033
Indirect effects								
TV →	Mindfulness →	Life Satisfaction	−0.081	0.035	−2.299	0.022	−0.150	−0.012
Total effects								
TV →	Life Satisfaction		−0.271	0.112	−2.420	0.016	−0.490	−0.052
Life satisfaction R2 = 0.043; Mindfulness R2 = 0.028.								

Note. Delta method standard errors, normal theory confidence intervals, ML estimator.

**Table 16:** Multiple regression model, the dependent variables are leisure preferences, and the predictors are life satisfaction, mindfulness, and psychological capital.

Dependent Variable	Predictors/ Models	Unstandardized Coefficients	Standardized Coefficients		*t*	Sig.	R	R2	Adjusted R2	F	Sig.
		B	Std. Error	Beta							
Spending time with Family	1 (Constant)	2.193	0.527		4.165	0.000	0.245	0.060	0.057	16.510 (1,259)	<0.001
	Life satisfaction	0.469	0.116	0.245	4.063	0.000					
Watching Television	1 (Constant)	5.095	0.958		5.321	0.000	0.178	0.032	0.028	8.428 (1,258)	0.004
	PsyCap relationship	−0.622	0.214	−0.178	−2.903	0.004					
Participating in Events	1 (Constant)	−0.385	0.961		−0.401	0.689	0.139	0.019	0.015	5.066 (1,259)	0.025
	PsyCap overall	0.480	0.213	0.139	2.251	0.025					

## Data Availability

The data that support the findings of this study are available from the corresponding author upon reasonable request.
